# Supplementary material for: Complexome profiling of the Chlamydomonas psb28 mutant reveals TEF5 as an early PSII assembly factor
Source: Plant Cell. 2025 Mar 17;37(6):koaf055. doi: 10.1093/plcell/koaf055 (PMC12164590; doi:10.1093/plcell/koaf055)
Supplement: koaf055_Supplementary_Data [file koaf055_supplementary_data.zip › psb28_interactive.zip!/plots/intensity/qualityPlots/peptides/MDN2_Cre10.g423250.t1.2.html]

### Cre10.g423250.t1.2

Trivial name: MDN2  
SeqCoverage [median]: 31.20  
Mass: 36.37 kDa  
  
 

| Sequence | MissedCleavage | Mass [Da] | wtIntensity | muIntensity | Score |
| --- | --- | --- | --- | --- | --- |
| AEAFIGELTGTDPR | 0 | 1475.73 | 882.32 | 0.00 | 76.52 |
| AGAGSATLSMAYAAAR | 0 | 1467.71 | 1767.04 | 0.00 | 149.49 |
| AMSGEGPVSEYAYIR | 0 | 1628.75 | 411.03 | 0.00 | 55.45 |
| DDLFNINAGIVR | 0 | 1345.70 | 1207.47 | 0.00 | 123.86 |
| IASHLDPAKPR | 1 | 1203.67 | 333.93 | 0.00 | 59.57 |
| IQDAGTEVVQAK | 0 | 1257.66 | 3869.65 | 1074.10 | 96.02 |
| LFGVTTLDVVR | 0 | 1218.70 | 1149.65 | 0.00 | 83.95 |
| VALLGAAGGIGQPLSLLLK | 0 | 1790.10 | 496.59 | 0.00 | 147.26 |
|  |  |  | 10117.7 | 1074.1 |  |
